# Supplementary material for: Effect of prebiotics on growth and health of dairy calves: A protocol for a systematic review and meta-analysis
Source: PLoS One. 2021 Jun 25;16(6):e0253379. doi: 10.1371/journal.pone.0253379 (PMC8232519; doi:10.1371/journal.pone.0253379)
Supplement: S1 Table — (DOCX) [file pone.0253379.s001.docx]

**S1 Table. PRISMA-P (Preferred Reporting Items for Systematic review and Meta-Analysis Protocols) 2015 checklist: recommended items to address in a systematic review protocol**

| Section and topic | Item No | Checklist item | Reported on page |
| --- | --- | --- | --- |
| ADMINISTRATIVE INFORMATION | | |  |
| Title: |  |  |  |
| Identification | 1a | Effect of prebiotics on performance and health of dairy calves: protocol for a systematic review and meta-analysis | 1 |
| Update | 1b | N/A |  |
| Registration | 2 | Systematic Review of Food Animals and Open Science Framework | 3 |
| Authors: |  |  |  |
| Contact | 3a | All names and institutional affiliations were provided and the e-mail of corresponding author | 1 |
| Contributions | 3b | The contributions of protocol authors were described | 1 |
| Amendments | 4 | Any amendments to this protocol will be documented and justified in the final review | 11 |
| Support: |  |  |  |
| Sources | 5a | The source of financial support for the review is indicated |  |
| Sponsor | 5b | This study was funded by the California Department of Food and Agriculture (CDFA) |  |
| Role of sponsor or funder | 5c | The funder had no role in the study design and in preparation of the manuscript |  |
| INTRODUCTION | | |  |
| Rationale | 6 | The rationale for the review was described. | 3 |
| Objectives | 7 | The research question and the objectives were explicit stated based on and the population, interventions, comparators, and outcomes (PICO) elements | 4 |
| METHODS | | |  |
| Eligibility criteria | 8 | The eligibility criteria were based on the PICO elements, language, publication type, and study design and a detailed description was provided | 5 |
| Information sources | 9 | The databases that will be used for searches were described as well as their coverage date | 6 |
| Search strategy | 10 | A description of the search strategy assembly was provided as well as a preliminary search | 7 and S3 |
| Data management | 11a | All software that will be used to manage records and data throughout the review were specified | 7 |
| Selection process | 11b | The criteria that will be used during screening for selection of abstracts and full-text manuscript were fully described as well as the reviewers that will execute it independently | 7 |
| Data collection process | 11c | The method of data extraction was described | 9 |
| Data items | 12 | All variables for which data will be sought were listed and defined | S2 |
| Outcomes and prioritization | 13 | The prioritization order of main and secondary outcomes was justified | 6 |
| Risk of bias in individual studies | 14 | All the domains of risk of bias that will be evaluated and its adjustments to fit animal science were described | 9 |
| Data synthesis | 15a | The criteria to perform a meta-analysis were defined | 10 |
|  | 15b | The methodology that will be used to assess heterogeneity was described | 10 |
|  | 15c | The planned additional analyses (sensitivity and subgroup analyses) were specified | 10 |
|  | 15d | If meta-analysis is unappropriated, studies characteristics and results will be descripted as a systematic review | 11 |
| Meta-bias(es) | 16 | The methodology that will be used to assess publication bias was described | 11 |
| Confidence in cumulative evidence | 17 | The methodology that will be used to assess the confidence in the evidence was described | 11 |
