# Supplementary material for: Effect of prebiotics on growth and health of dairy calves: A protocol for a systematic review and meta-analysis
Source: PLoS One. 2021 Jun 25;16(6):e0253379. doi: 10.1371/journal.pone.0253379 (PMC8232519; doi:10.1371/journal.pone.0253379)
Supplement: S2 Table — (DOCX) [file pone.0253379.s002.docx]

**S2 Table. Definition of main and secondary outcomes**

| **Outcome** | **Definition** | **Reference** |
| --- | --- | --- |
| Average daily gain | rate of live weight gain per day over a specified period | [1] |
| Feed efficiency | ratio of feed intake to live weight gain | [1] |
| Body traits | indirect method to estimate the body weight | [2] |
| Heart girth | circumference of the animal just behind the withers | [2] |
| Body length | distance from the point of the shoulders to the ischium | [2] |
| Hip width | distance between the left and right [greater trochanters](https://www.sciencedirect.com/topics/veterinary-science-and-veterinary-medicine/greater-trochanter) of femurs | [2] |
| Wither height | distance from the floor beneath the calf to the top of the withers directly above the center of the shoulder | [2] |
| Dry matter intake | amount of feed consumed per day on a moisture-free basis | [3] |
| Papilla length | distance from the tip to the base of the papilla along its axis | [4] |
| Papilla width | measure of half of the perpendicular papilla length | [4] |
| Ruminal pH | hydrogen ion concentration in rumen contents | [5] |
| Volatile fatty acid concentration | concentration of acetate, propionate, and butyrate in the ruminal fluid | [6] |
| Beta-hydroxybutyrate | measurement of the concentration of this ketone body in blood | [7] |
| Glucose | measurement of the concentration of this hexose in blood | [7] |
| Immunoglobulin | measurement of the concentration of IgG, IgA, IgM, or IgE in blood | [8] |
| Fecal score | visual evaluation of the fecal consistency; indirect indicator of the severity and the presence of diarrhea | [9] |
| Diarrhea incidence | proportion of the calves identified as diarrheic within a specified period | [10] |
| Mortality | number of calf deaths during a specific time | [11] |
| Cytokines | concentration of signaling proteins that can be pro- or anti-inflammatory in blood | [12] |

1. Berry DP, Crowley JJ. Residual intake and body weight gain: A new measure of efficiency in growing cattle. J Anim Sci. 2012;90: 109–115. doi:10.2527/jas.2011-4245

2. Wilson LL, Egan CL, Terosky TL. Body Measurements and Body Weights of Special-Fed Holstein Veal Calves. J Dairy Sci. 1997;80: 3077–3082. doi:10.3168/jds.S0022-0302(97)76277-5

3. NRC. Nutrient Requirements of Dairy Cattle. 2001. 7th rev. ed. Natl. Acad. Sci., Washington, DC.

4. Dieho K, Bannink A, Geurts IAL, Schonewille JT, Gort G, Dijkstra J. Morphological adaptation of rumen papillae during the dry period and early lactation as affected by rate of increase of concentrate allowance. J Dairy Sci. 2016;99: 2339–2352. doi:10.3168/jds.2015-9837

5. Marden JP, Bayourthe C, Enjalbert F, Moncoulon R. A new device for measuring kinetics of ruminal pH and redox potential in dairy cattle. J Dairy Sci. 2005;88: 277–281. doi:10.3168/jds.S0022-0302(05)72685-0

6. Yohe TT, Schramm H, White RR, Hanigan MD, Parsons CLM, Tucker HLM, et al. Form of calf diet and the rumen. II: Impact on volatile fatty acid absorption. J Dairy Sci. 2019;102: 8502–8512. doi:10.3168/jds.2019-16450

7. Knowles TG, Edwards JE, Bazeley KJ, Brown SN, Butterworth A, Warriss PD. Changes in the blood biochemical and haematological profile of neonatal calves with age. Vet Rec. 2000;147: 593–598. doi:10.1136/vr.147.21.593

8. Butler JE. Bovine immunoglobulins: An augmented review. Vet Immunol Immunopathol. 1983;4: 43–152. doi:10.1016/0165-2427(83)90056-9

9. Larson LL, Owen FG, Albright JL, Appleman RD, Lamb RC, Muller LD. Guidelines Toward More Uniformity in Measuring and Reporting Calf Experimental Data. J Dairy Sci. 1977;60: 989–991. doi:10.3168/jds.S0022-0302(77)83975-1

10. Ma FT, Wo YQL, Shan Q, Wei JY, Zhao SG, Sun P. Zinc-methionine acts as an anti-diarrheal agent by protecting the intestinal epithelial barrier in postnatal Holstein dairy calves. Anim Feed Sci Technol. 2020;270: 114686. doi:10.1016/j.anifeedsci.2020.114686

11. Santman-Berends IMGA, Schukken YH, van Schaik G. Quantifying calf mortality on dairy farms: Challenges and solutions. J Dairy Sci. 2019;102: 6404–6417. doi:10.3168/jds.2019-16381

12. Murtaugh MP, Foss DL. Inflammatory cytokines and antigen presenting cell activation. Vet Immunol Immunopathol. 2002;87: 109–121. doi:10.1016/S0165-2427(02)00042-9
