# Supplementary material for: Effect of prebiotics on growth and health of dairy calves: A protocol for a systematic review and meta-analysis
Source: PLoS One. 2021 Jun 25;16(6):e0253379. doi: 10.1371/journal.pone.0253379 (PMC8232519; doi:10.1371/journal.pone.0253379)
Supplement: S3 Table — (DOCX) [file pone.0253379.s003.docx]

**Table 1. Preliminary electronic search string used to retrieve studies examining supplementation of prebiotics for dairy calves on October 28^th^ of 2020**

|  |  | **PubMed** | **Cab Direct** | **Scopus** | **Web of Knowledge** | **ProQuest** |
| --- | --- | --- | --- | --- | --- | --- |
| **ID** | **Terms** | **Results** | | | |  |
| #1 | “Calf” OR “calves” [tiab] OR “veal” [tiab] OR “preweaned dairy heifers” [tiab] | 65,827 | 103,737 | 97,327 | 34,758 | 5,695 |
| #2 | "Prebiotics"[Mesh] OR “dietary fiber”[tiab] OR “prebiotics”[tiab] OR OR “short-chain fatty acid”[tiab] OR “conjugated linoleic acid”[tiab] OR “conjugated linoleic acids”[tiab] OR “CLA”[tiab] OR “PUFA”[tiab] OR “polyunsaturated fatty acid”[tiab] OR “polyunsaturated fatty acids”[tiab] OR “fructooligosaccharides”[tiab] OR “polysaccharide”[tiab] OR “polysaccharides”[tiab] OR “galactooligosaccharide”[tiab] OR “galactooligosaccharides”[tiab] OR “xylooligosaccharides”[tiab] OR “xylooligosaccharide”[tiab] OR “oligosaccharides”[tiab] OR “mannanoligosaccharide”[tiab] OR “mannanoligosaccharides”[tiab] OR “glycans”[tiab] OR “inulin”[tiab] OR “phenolic acid”[tiab] OR “phenolic acids”[tiab] OR “phenolics”[tiab] OR “volatile fatty acids”[tiab] OR “volatile fatty acid”[tiab] OR “FOS”[tiab] OR “MOS”[tiab] OR “cellooligosaccharide”[tiab] OR “cellooligosaccharides”[tiab] OR "Dietary Supplements"[Mesh] OR “tannins”[tiab] OR “flavonoids”[tiab] OR “tannin”[tiab] OR “lignans”[tiab] OR “neolignans”[tiab] OR “Phytotherapy/veterinary”[Mesh] OR “Plant Extracts/therapeutic use”[Mesh] OR “Fatty Acids, Volatile”[Mesh] OR “Tannins/therapeutic use”[Mesh] OR "Polysaccharides"[Mesh] OR "Linoleic Acids, Conjugated/therapeutic use"[Mesh] OR "Polysaccharides/therapy"[Mesh] OR "Polysaccharides/veterinary"[Mesh] OR "Fatty Acids, Unsaturated/therapy"[Mesh] OR "phenolic acid" [Supplementary Concept] OR "Lignans/therapeutic use"[Mesh] | 950,385 | 408,060 | 1,136,592 | 181,503 | 35,368 |
| #3 | “Fecal score”[tiab] OR “faecal score”[tiab] OR “weight gain”[tiab] OR “feed efficiency”[tiab] OR “diarrhea”[tiab] OR “diarrhoea”[tiab] OR “diarrheal”[tiab] OR “diarrhoeal”[tiab] OR “scours”[tiab] OR “scouring”[tiab] OR “intestinal development”[tiab] OR “growth”[tiab] OR “health”[tiab] OR “mortality”[tiab] OR "Diarrhea/microbiology"[Mesh] OR "Diarrhea/mortality"[Mesh] OR "Diarrhea/veterinary"[Mesh] OR "Cattle/growth and development"[Mesh] OR "Weight Gain"[Mesh] | 4,160,541 | 2,286,014 | 10,542,504 | 939,887 | 526,512 |
| #4 | #1 AND #2 AND #3 | 1,181 | 1,023 | 1,295 | 55 | 43 |
